# Address before the Convention of Delegates from Medical Colleges

**Published:** 1870-05

**Authors:** David W. Yandell

**Affiliations:** Prof. of Clinical Surgery in the University of Louisville, Ky.


BUFFALO

### Medical and Surgical Journal.

VOL. IX. MAY, 1870. No. 10.

### Original Communications.

ART. I.—*Address before the Convention of Delegates from Medical Colleges. Held in Washington, May 2d,* 1870. By DAVID W. YANDELL, M. D., *Prof. of Clinical Surgery in the University of Louisville, Ky.*

MR. PRESIDENT:

I beg to ask here just two questions: Can the recommendations of the committee be carried out so as to be made effective? Is it expedient to carry them out? The first inquiry I, myself, answered unhesitatingly that there does not exist to-day, in this country, anywhere a power which can make such requirements obligatory or binding upon any person. There is, in my judgment, no authority delegated to, or resident in anybody, by which a single student of medicine in all this land can be compelled to possess the preliminary knowledge and acquirements set forth in the resolution now under consideration. And unless such a power exists, unless we can be invested with some such authority, why go again through the empty farce of committing ourselves to it? Delegates have addressed this Convention as though we, the teachers, the representatives of the schools, had nothing further to do than to issue a kind of pronunciamento touching the preliminary acquirements of students, when lo! every applicant for our tickets would henceforth come as a Latinist, as well grounded in the Greek, and strong in the higher mathematics. My distinguished friend, Prof. Hammer, of Missouri, has indulged in even more than the usual amount of declamation about the honor, and glory, and dignity, and usefulness, and beneficence of our noble profession. He has paid the accustomed tribute to the all-pervading sun of science, and altogether has done that part of the business in a most admirable way. He would have all doctors most learned pundits—all students of medicine finished scholars before they could enter the sacred temple of the Gods of Medicine, or enlist, even in the humblest capacity, in the ranks of the mighty army which is so unceasingly advancing on the great enemies of our race—disease and death. All of us have heard very much this same talk many times before. Most of us have indulged in it more or less. It has, I suppose, its uses. It makes up the staple of a large number of introductory lectures, and is generally thought to be popular with the public. We, down in my country, call it *gush*. Another word for it is *bosh*. I know some very clever people who think teachers have gushed quite enough on these topics. The war did the business for *gush* among most of my people. We have abandoned it. We have come now to take things as they are; we *gush* about them no more. We take people as we find them. We take the world as it is. We regard that unfortunate class of our fellow citizens, which anticipates becoming our successors in legitimate medicine, as being just as good as we were when we stood where they stand to-day. I know this isn't just now the fashionable idea, but it is the one we hold to in Kentucky These young men who are to succeed us are the children of our friends, of our associates, our neighbors, our patrons, our equals. We even believe they know as much as we did when we began. Nay, more; we actually expect that they will know more than we do now when they come to be as old as we now are. Their attainments are as varied, as solid, and in every sense as respectable, as ours were. Their zeal is as noticeable, and their ambition is as great, as ours were. We were driven to admit that they are even as good as we were. Yet, forsooth, we are gravely told by certain delegates, that because these unfortunates are not *more* learned than we were; because they cannot do what the fewest of us could do when we began, what many of us cannot do now; because they have no knowledge of the Greek, and are ignorant of the Latin, and unfamiliar with the higher mathematics, they must be denied admission into our colleges; that, because the country doesn't produce classical scholars who wish to study medicine, schools must cease to make doctors. And the several medical institutions now in our land are urged, with some very lofty airs, to close their doors on all such young men as are deficient in these, as we are told, the essentials of a professional education. I should like to ask you, Mr. President, how many teachers of your acquaintance are competent even to examine students in the branches laid down in this resolution, which have so suddenly grown to be so nearly indispensable to admission into our schools? How many of our professors of medicine, do you believe, can read a sentence in Greek, or a page in Latin? All this talk about classical learning is the sheerest stuff; and in dwelling upon it with such tenacity, we but show ourselves to the world a pack of solemn shams. There isn't one of us who doesn't know that an acquaintance with the dead languages is not *necessary* to the completest understanding of the diseases of the living, teeming people of the world. Great physicians, consummate masters of the healing art, men sought after because of their unequalled skill, from all parts of the land, there are to-day, and will be, I doubt not, a hundred years hence, who don't know the Greek alphabet, and couldn't, to save their necks, parse a line of Latin. I will not detain you by citing individual instances of men, who, having shed lustre upon all of medicine, have been gathered to their reward in happy ignorance of the classics. They will occur to the minds of my hearers. Nor have I occasion to go further than this audience to establish the truth of what I say. When you and I, Mr. President, selected the profession of medicine as our calling, could either of us have engaged in its study had this resolution been in force? Could you read Greek? Were you familiar with Latin? Had you gone any great distance in the mathematics? And yet, sir, you have succeeded beyond most men. All the honors within the gift of the profession have been heaped upon you; to-day you stand, confessedly, at the head of American Surgery. You have been this moment elected President of all these learned Thebans. Must you turn now, and exact of the student what you, yourself, did not possess at his age, "a sufficient knowledge of the Greek and Latin to enable him to understand the technical terms of the profession?" Why, sir, if this sufficient knowledge doesn't mean a thorough knowledge of those languages, then it means nothing. And besides all this, the embryonic Esculapius must be conversant with mathematics. I beg to offer the following problem to my friends of a mathematical turn of mind. If this resolution be adopted and could be enforced, how many students would there be in attendance upon lectures the coming winter, and how many teachers would there be capable of examining them in these prerequisites of medical education? I fancy there would be proportionally as large a falling off among the teachers as the taught. Again: Would medical education cease, and no more of these ignorant youngsters—who are such bug-bears to some of my learned friends—be turned loose upon the world? Not at all. The same law which controls other matters in this world, regulates the supply and quality of doctors. The old law of supply and demand applies to physicians, just as to other men and other things; nor can we reverse it. What the people want, the people will have, the people will finally get. If they want physicians skilled in medicine and learned in the classics, such physicians will be produced. But, I ask, *do* our people demand such? If so, I should be glad to see the evidence of the fact. If the mathematical wing of this convention be in possession of it, let them produce it. If such physicians are demanded, why is the material for their production not furnished? The schools can use only such material as is sent them. The schools can't require of their pupils a degree of scholarship in advance of their surroundings. The schools cannot demand of their students classical attainments beyond and above those exacted by the law and the ministry. Why may we not profit by the practice of the courts in this matter? The student of the law applies to the courts for license to practice his profession. The learned court examines the trembling aspirant on the law and the practice, not on Cæsar and Xenophon, and if his answers be satisfactory, he is invested with the coveted degree. Shall we do more? Are we better than they? I have already said that the schools have no power to say, "You shall know Greek, and Latin, and mathematics, or you shall neither study nor practice medicine." The coming doctor would snap his finger in your face for your pains. And if the schools *had* the power, to say that they would exercise it, is but another bit of *gush*, a huge piece of *bosh*, so exceedingly transparent that it deceives no one, not even ourselves. The man who is sick pays his money for professional skill, not for classical lore. The surgeon who reduces a dislocation most deftly; who is tenderest, gentlest, firmest, and handiest; the physician who is the most careful, most observant, most sagacious, most sympathetic, and promptest, will always win in the long run, though he may not know a Greek character, or own a Latin grammar.

Yet, while I say all this, let no one charge that I undervalue scholastic attainments, or think lightly of preliminary education. I should indeed be glad to see every physician learned in the classics, as well as in medicine. We have authority for believing that he would be happier—that it would soften his manners, and refine his tastes, and increase his dignity. And I do not deny it. "But the old trouble still presses upon us;" art is long, life is short. Year by year medicine extends its boundaries, and each succeeding conquest but adds to the difficulty of making ourselves masters of the situation. To read the books which to-day are published on a single branch of surgery alone, would require more time than was needed, fifty years ago, to get through a complete medical library.

Again: After all that has been said about preliminary education, are you, yourselves, agreed either as to what, or how much, the student shall know before he can enter our colleges? By no means. Your opinions are almost as diverse as the States you represent. One teacher proposes to omit the Greek. Another, equally experienced, suggests that a knowledge of neither the Latin nor Greek be required, but holds fast to the mathematics. Another declares that he would be content if the medical student could have a sound English education, while my distinguished friend from Maryland is almost persuaded that the "*examen rigorosum*" of the University of Berlin should be instituted in our colleges.

Sir, the profession in this country is a law unto itself. It needs not to cross the Atlantic to learn its wants, or how to supply them. The rules which govern the medical student in Europe cannot yet, at least, be applied to the student in America The "*examen rigorosum*" of Prussia may, and doubtless does, tend to make most erudite physicians. It has yet to be shown that it makes practitioners who are more successful than the unfortunate persons who get their diplomas from our much abused colleges. When my excellent friend has been longer engaged in his new calling, that of editing a medical journal, he will realize that all the scholarship of the profession will not die with the professors; and it will not be long before he will be glad to get communications for his admirable periodical, written by teachers, in even passable English. He won't have it in his kindly heart to apply the "*examen rigorosum*" to their articles, though he is half inclined to set it up for the student.

I have another problem for the mathematical section of this convention: If this system, under which we are working, is so defective, and so altogether abominable, how is it that we have so many great doctors throughout the country? How does it come to pass that we have such an array of renowned teachers here to-day? With a single exception every one of us is a graduate of an American medical college. But one, of all this grave and learned assembly, has a European diploma—but one has undergone that rigorous examination of which we have heard, and yet I feel safe in saying that each and every one of us enjoys the respect, the confidence, and the esteem of the community in which we live. Otherwise we should not be here.

We have been threatened by some of the more enthusiastic of our members, that unless this convention do something, the National Medical Association, which is to meet here next week, will take the matter in hand, and do the business for us. I wish it would. I should like to see how it would go about it. The National Medical Association has its work. We have ours. A year ago we were warned that, if in no other way, the necessary reforms would be effected by "an uprising of the people." The time for this interesting event has not been fixed. All I have to say concerning it is, that when the uprising occurs it won't get very high, or last very long. The people will soon get back to employing the doctor that suits them the best, without being over-nice as to what diploma he has. Communities have quite a summary way of disposing of such matters. If they don't like the doctors in their midst, they import others or seek, elsewhere, the aid they require. I hold that it is not on the people, but on itself that the profession must rely. This convention is helping it. The Medical Association is helping it. And if "this great society will go on in the path it has been pursuing, trusting for results to its wise suggestions and its incidental influence, and never turning its thoughts to legislation, it will yet do much more for the profession." The moment that it forsakes this policy it may bid adieu to its power; the moment that it assumes the role of a law-giver, it will cease to be an influence. It may advise. It may suggest. It may urge. Let it not attempt to compel. This restless, driving, pushing, swift, all pervading Anglo-American people would not brook, for a single instant, any such compulsory legislation. When one of its number decides on medicine as a calling, he will begin its study. In due time he will apply at your colleges. If you refuse him admission, nothing daunted, not even in the least disconcerted, he will buy the books he wishes, and when he has thumbed them as long as he thinks necessary, he will tack up his shingle, prefix the title of doctor to his name, and practice on whomever chooses to employ him. Nor have we, the teachers, nor has the National Medical Association, any means to prevent or hinder him. To acquire such power would demand a change in the organic law of the land so radical, so utterly revolutionary, that even the most Utopian of us all would hardly dare hope for it. Then why talk about it? Why meet, year after year, and try anew, schemes that every practical man here knows are impracticable. I am unwilling to countenance such wholesale legislation; because it is useless, because it is absurd. Haven't recommendations and resolutions, touching all this subject, been annually repeated by the National Medical Association for nearly a quarter of a century, to be disregarded just as often and so long by some of the leading colleges of the country?

I have just here another problem for the mathematicians. If twenty-two years have been exhausted in the fruitless endeavor to induce certain leading colleges in the East to adopt the simplest suggestions of the National Medical Association, how many years will it require to educate the medical students of the country up to the standard of the resolution we are considering? If, as I state, some of the colleges of the East pay no heed to the oft-repeated wishes of the Association, shall those of the West be required to do so? Every one here present must be aware that if the Western schools had endeavored to enforce the resolutions of the Association, many of their students would have found their way to the East. Starting from beyond the Mississippi they would, when denied admission at St. Louis, because they fell short of the necessary requirements, have gone to other western schools to meet the same fate. Does any one here believe they would have gotten further East than Philadelphia? Do you think, sir, they would by any possibility have found their way through the great State of Pennsylvania to the city of New York?

But, Mr. President, to change the style of my remarks, and bring them to a close, I beg to suggest a platform on which we may all unite. I propose that we give more thought to the teachers, and less to the taught; more thought to the character of the schools, and less to the scholars. There are many thousands of the latter. Instead of inveighing against the ignorance of the pupil, let us improve the scholarship of the professor. Finally, let us all endeavor at least to live up to the requirements which have been so long upon the statute-books of the National Medical Association, and nominally accepted by all the faculties of the Union. It will be something to do that. When we all, in good faith, take the first step, the rest may not be so difficult.

